# *In silico* modelling of mass transfer & absorption in the human gut

**DOI:** 10.1016/j.jfoodeng.2015.10.019

**Published:** 2016-05

**Authors:** T.E. Moxon, O. Gouseti, S. Bakalis

**Affiliations:** Chemical Engineering, The University of Birmingham, Edgbaston, Birmingham, B15 2TT, UK

**Keywords:** *In silico* modelling, Human gut, Glucose absorption, Mass transfer, Gastric emptying

## Abstract

An *in silico* model has been developed to investigate the digestion and absorption of starch and glucose in the small intestine. The main question we are aiming to address is the relative effect of gastric empting time and luminal viscosity on the rate of glucose absorption. The results indicate that all factors have a significant effect on the amount of glucose absorbed. For low luminal viscosities (e.g. lower than 0.1 Pas) the rate of absorption is controlled by the gastric emptying time. For viscosities higher than 0.1 Pas a 10 fold increase in viscosity can result in a 4 fold decrease of glucose absorbed. Our model, with the simplifications used to develop it, indicate that for high viscosity luminal phases, gastric emptying rate is not the controlling mechanism for nutrient availability. Developing a mechanistic model could help elucidate the rate limiting steps that control the digestion process.

## Highlights

• The stomach and small intestine were modelled as ideal reactors.
• Absorption of glucose was modelled as a function of viscosity within the lumen.
• Increasing the viscosity reduced the amount of glucose absorbed.
• The gastric emptying rate is an important factor in the absorption of nutrients.

## 1 Introduction

Understanding digestive processes is important in addressing diet related diseases, such as obesity, which are becoming a major problem all around the world. A World Health Organisation report in 2014 stated that 39% of adults were overweight and 13% were obese; also stating that the obesity rate was most prevalent in the Americas and least in the south-east Asian regions (WHO, 2014). Specifically in the UK around a quarter of adults were classified as obese as of 2014 (HSCIC, 2014); it has been estimated that obesity will cost the UK society £50billion per annum by 2050 (McPherson et al., 2007). In order to address some of the food related diseases and design healthier foods it is important to understand the behaviour of foods during digestion using *in silico* as well as *in vivo* and *in vitro* studies.

Modelling has been extensively used in a variety of systems e.g., pharmaceuticals (Peng and Cheung, 2009, Stoll et al., 2000), biological systems such as the insulin-glucose system (Makroglou et al., 2006, Pedersen and Cobelli, 2014). Simulation of biological processes allows for investigation into phenomena that are difficult to examine or study *in vivo* and *in vitro*. In this work we will be modelling digestion in the gut as a series of ideal reactors, a concept introduced in the late 1980's (Penry and Jumars, 1986, Penry and Jumars, 1987), with wide applications in the area of pharmacokinetics (Ni et al., 1980, Peng and Cheung, 2009, Stoll et al., 2000).

Mathematical models have been developed to investigate the digestion of foods using different approaches: A compartmental approach with a CSTR small intestine was used by Dalla Man et al. (2006) assuming that changes in gastric emptying rate have the largest effect on absorption (Dalla Man et al., 2006), this work showed good agreement with absorption from oral glucose tolerance tests. Bastianelli et al. (1996) simulated the movement and absorption of different nutrients simultaneously with a multiple compartmental approach (Bastianelli et al., 1996), which was able to predict nutrient absorption patterns and transit times. A model developed by Taghipoor et al. (2012) used a system of ODEs to simulate the movement and absorption from a food bolus within the intestine highlighting the effect dietary fibre has on slowing the bolus break down (Taghipoor et al., 2012, Taghipoor et al., 2014).

Despite the fact that mathematical models provide insight into digestion; they typically use parameters that are obtained empirically, which limits their predictive capability.

### 1.1 Starch digestion

Starch is the largest source of carbohydrate in the human diet (Singh et al., 2010). In the small intestine, α-amylase will convert starch to oligosaccharides, and brush boarder enzymes (e.g., glucoamylase) will hydrolyse the oligosaccharides to glucose, which can then be absorbed. The conversion of oligosaccharide to glucose and absorption of glucose by sodium-dependent glucose cotransporter 1 (SGLT-1) proteins through the epithelium will be rapid and will not be rate limiting (Bastianelli et al., 1996, Lentle and Janssen, 2011, Stumpel et al., 2001).

The kinetics of starch hydrolysis by α-amylase has been studied by a number of authors with the amylase substrate isolated from a variety of sources. Both bacterial and human α-amylase have been found to follow Michaelis–Menten kinetics (Ikram-Ul-Haq et al., 2010, komolprasert and Ofoli, 1991, Satomura et al., 1984, Yankov et al., 1986), although it has also been reported that this is only followed for low substrate concentrations and at high concentrations a modified 1st order kinetics can be used (komolprasert and Ofoli, 1991). Inhibition of α-amylase by high d-glucose concentrations has been reported on some occasions (Steverson et al., 1984, Yankov et al., 1986), which has been reported to have a large effect at concentration greater than 300 g/L (Yankov et al., 1986), though this is a high concentration that is unlikely to be encountered *in vivo.*

### 1.2 Gastric emptying

Gastric emptying rate is often considered to be the rate limiting step in the absorption of nutrients (Hellstrom et al., 2006, Mourot et al., 1988). The delivery of gastric content to the duodenum is controlled by the pyloric sphincter (Hellstrom et al., 2006), whilst the stomach acts as a reservoir for consumed food, and mechanically and chemically breaks down the content (Kong and Singh, 2008).

Table 1 shows a selection of studies of the gastric emptying rate for different liquid solutions. Gastric emptying is quantified with a half-time (time for half the content to empty the stomach by volume) and calorific empting rate. These studies were selected as they have a comprehensive description of the physical properties of the fluids and the calorific content.

**Fig. 1:**
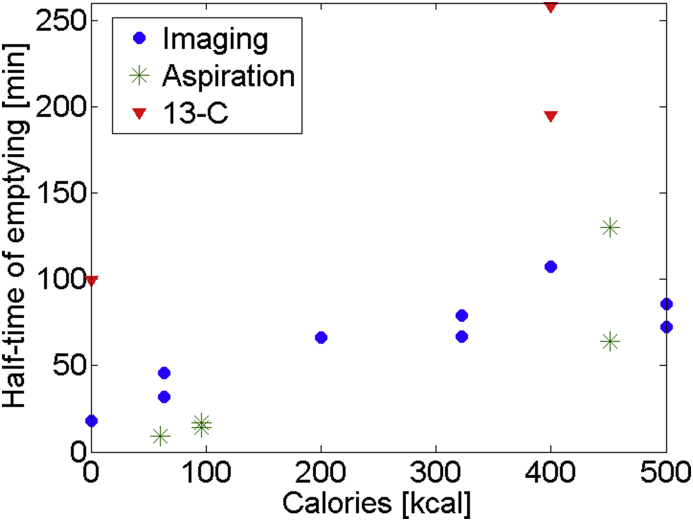
plot of half-time of emptying against calories for meals in Table 1, different colours represent different methods of measurements, showing that increasing the calorific content of a meal leads to a longer half time of emptying.

In Table 1 the measurement methods can be separated in 3 groups: breath sampling, aspiration, and imaging (e.g. MRI/Scintigraphy/Sonography). The most common method for measuring gastric emptying rates in a medical setting is Scintigraphy, where meals are labelled with ^99m^Tc, and distributions of these radioisomers are taken using gamma cameras (Punkkinen et al., 2006). Punkkinen et al. (2006) compared this to the ^13^C breath test, where a meal is labelled with ^13^C and breath samples are taken and the ratio of ^13^C–^12^C can be used to calculate the volume remaining in the stomach. The group found that the ^13^C breath test gives significantly longer emptying half-time than Scintigraphy and that there was no correlation between the half-lives of the two methods (Punkkinen et al., 2006). This could explain why the results by Shimoyama et al. (2007) have longer emptying rates when compared to the rest of the table (also shown in Fig. 1).

Scintigraphy has also shown 70% slower emptying rates than double sampling aspiration, where a dye is added to a meal and samples are taken directly from the stomach via catheter and emptying inferred (Beckers et al., 1992), although this is not evident from the data presented in Table 1. Good agreement in measured emptying rates with MRI (Feinle et al., 1999, Schwizer et al., 1992) and ultrasonography (Hveem et al., 1996) are also shown in literature.

Fig. 1 shows a plot of half-time of emptying against the calorific content of the meal for different measurement methods. As one can see the resulting emptying times depend on the method of measurement. As previously explained the ^13^C method results in a significantly higher estimation of gastric emptying time; this results in a large uncertainty on parameters used in models as a large variety of sources have to be considered typically each employing a different method.

As can be seen in Fig. 1, an increase in calorific content results in an increase of gastric emptying time, but the scarcity of the data points do not allow us to conclude upon the nature of the relationship. This could be explained from observations widely reported in literature of a feedback mechanism from the small intestine (controlled by nutrient sensors) that is thought to be the main controlling mechanism of gastric emptying rate (Brener et al., 1983, Calbet and MacLean, 1997, McHugh, 1983, Shimoyama et al., 2007).

Whilst in Table 1 there is a clear trend with emptying rate and the calorific content, the link between the emptying rate and viscosity or volume of meal consumed is not clear. Prior to the initiation of this feedback mechanism, there is an initial rapid emptying rate, which is independent of the nutrient content of the meal. Some researchers suggest that this rate will be controlled by the volume of fluid in the stomach (Brener et al., 1983, Moran et al., 1999), while others point to the effect of viscosity (Marciani et al., 2001, Shimoyama et al., 2007), with more viscous meals causing greater distension of the antral region relative to the proximal; and also resulting in a great volume of gastric secretions (Marciani et al., 2001). However, contradictory results on the effect of viscosity on gastric emptying have been reported, as seen in Table 1. The effect of gastric secretions could play a key role in determining gastric viscosity (see for example (Marciani et al., 2000)).

The last two results in Table 1 indicate the difference in emptying between two meals of the same constitution, one in solid/liquid form and one as a soup. There is a difference between how solids and how liquids will empty from the stomach, with solids requiring a reduction in particle size, to around 1–2 mm, before they can empty (Hellstrom et al., 2006). The current work will focus on the ingestion of liquid meals and the gastric processes will not be considered, other than the emptying to the small intestine.

### 1.3 Modelling of absorption in the small intestine

Within the intestinal lumen the chyme (mixture of consumed food and secretions from the digestive system) will be propelled aborally and via peristaltic contractions, which may also provide mixing of the nutrients (Janssen et al., 2007). Segmentation contractions will mix the chyme with no movement axially along the intestine (Ganong, 2005). The flow of nutrients along the digestive tract has been studied by numerous authors using computation fluid dynamics (CFD). Studies have been carried out to look at the mixing effects in the stomach (Ferrua and Singh, 2010, Ferrua and Singh, 2011, Kuzo et al., 2010), the flow at the gastroduodenal junction (Dillard et al., 2007), and the flow in the intestine (Love et al., 2013, Nadeem et al., 2012, Riahi and Roy, 2011, Tripathi, 2011, Tripathi et al., 2011). These studies indicate that flow dynamics will affect the movement of nutrients to the luminal wall; this mass transfer can be an important parameter in nutrient bioaccessibility (whether the nutrients are in a form which can be absorbed).

*In silico* (computer simulated) studies of absorption in the small intestine have been carried out for drug and foods using different methodologies. In pharmacokinetics, two main types of models have been used: non-compartmental and compartmental. Non-compartmental models are generally developed by fitting a mathematical expression to *in vivo* data, hence the fitted parameters will be accurate only for the system analysed and will not offer any predictive capability. In compartmental models, the system is divided in to compartments each representing a different physiological process; each with different mathematical expressions. A well formulated model should offer a certain amount of predictive capability (Peng and Cheung, 2009).

In literature the small intestine has been modelled as a single compartment (Dalla Man et al., 2006, Di Muria et al., 2010), as multiple compartments (Bastianelli et al., 1996, Yu et al., 1996) or as a plug flow reactor (PFR) (Logan et al., 2002, Stoll et al., 2000).

The model developed by Dalla Man et al. (2006) attempted to simulate an oral glucose tolerance test and a test meal. The intestine was modelled as a continuous stirred tank reactor (CSTR) with an input from the emptying of the stomach which is a function of the mass of glucose in the stomach. The model required the fitting of 6 parameters and had overall a good agreement with experiments (p < 0.005). However, a more physiologically relevant control for gastric emptying is the sensing of nutrients in the duodenum, followed by the relevant feedback response (Brener et al., 1983, Calbet and MacLean, 1997). In addition, there is no consideration of food properties, which are likely to affect absorption (Gouseti et al., 2014, Tharakan et al., 2010), this will be one of the focus of the models developed in this work.

Yu et al. (1996) compared different compartmental and plug flow models, concluding that the flow profile in the small intestine can be characterised with both a multi-compartmental model and a plug flow model, but that a single-compartmental model, as used by Dalla Man et al. (2006) was inadequate at describing the flow profile.

A multi-compartmental model was developed by Bastianelli et al. (1996) for a meal containing a variety of nutrients; the model used 4 compartments described by a series of ordinary differential equations. Although successful in describing digestion of a complex meal, the model does not consider the effect of one component on another (e.g. the effect of fibre on bioaccessibility of nutrients), the effect of nutrients on stomach emptying, nor the spatial location or movement along each compartment (Bastianelli et al., 1996). Another method, used by Stoll et al. (2000), was a plug flow model for the absorption of drugs in the small intestine. This model included the effect of increased surface area due to the folds and projections on the surface of the small intestine, and the effect of eddy rolls, to give good agreement with the absorption and degradation within the systemic circulation system (Stoll et al., 2000). The model does not include any gastric disintegration or emptying effects, which might have significant implications on the absorption of nutrients.

The movement and degradation of a bolus in the small intestine was investigated by Taghipoor et al. (2012). The model considered the effect of non-degradable and soluble nutrients; it was further developed to look at the effect of dietary fibre (Taghipoor et al., 2014). This will have an effect on the viscosity and water holding capacity of the bolus, and on the absorption of nutrients (Taghipoor et al., 2014). This highlights the importance of bioaccessibility during digestion.

An all-in-one model would allow full representation of physiological conditions, however this will be at the expense of simplicity, increase the difficulty of implementation and requiring a large number of parameters, with doubtful advantages over *in vivo* tests. The different factors need indeed to be considered separately, and with their relative importance (Calbet and MacLean, 1997, Hellstrom et al., 2006, McHugh, 1983).

In this paper a mechanistic approach to the modelling of mass transport and absorption from the small intestine is attempted; focusing on the effect of the delivery of the nutrients to the small intestine from the stomach, the mass transfer (as a function of viscosity of the chyme) within the lumen of the small intestine and the hydrolysis of starch prior to absorption. This study will focus on the use of a plug flow reactor small intestine, assuming a laminar flow and constant mean velocity. In reality the regime of flow will depend on the nature of the chyme; lower viscosity solutions exhibiting turbulent flow, and more viscous solutions displaying laminar flow with large regular vortices (Janssen et al., 2007, Lentle and Janssen, 2008), as a result of wall contractions and curvature of the small intestine. Hence the assumption of laminar flow will likely underestimate the mass transfer of nutrients at particular viscosities and also affect the residence time distribution (Janssen et al., 2007), but the comparison of this parameter to the gastric emptying rate, and hydrolysis rate should still be revealing.

This paper will present three models of increasing complexity. In the first model, mass transfer of nutrients (exemplified by glucose) within the small intestine and through the intestinal wall will be linked with the viscosity of chyme. The effect of gastric emptying on glucose absorption will then be considered in the second model. The third model will include starch hydrolysis, assuming the reaction to follow Michaelis–Menten kinetics.

## 2 Development of models

The following models (models 1–3) have been developed to investigate different factors that could influence the absorption of nutrients: bioaccessibility within the small intestine, gastric emptying rate and hydrolysis rate.

**Fig. 2:**
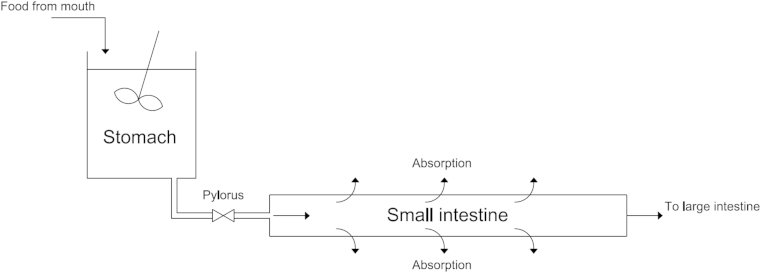
diagram showing layout of CSTR stomach and PFR small intestine.

The models assume that the stomach and small intestine can be described by a series of reactors, specifically a continuous stirred tank reactor (CSTR) for the stomach, which will act as a reservoir and control the emptying of contents only, and a plug flow reactor (PFR) for the small intestine (Fig. 2).

The models will be developed with increasing complexity, the first looking at the effect of mass transfer within the lumen on absorption of nutrients; the next will include the mass transfer and gastric emptying rate and how these both affect the absorption rate; the final model will look at, mass transfer rate, gastric emptying rate and starch hydrolysis, and how all 3 effect the absorption of nutrients.

### 2.1 Model 1: glucose absorption

This first model aims to investigate the effect of mass transfer on glucose absorption in the small intestine; this was modelled as a 1D advection-reaction equation (Logan et al., 2002):

(1) $$\frac{\partial G\left(z,t\right)}{\partial t}=-\bar{u}\frac{\partial G\left(z,t\right)}{\partial z}-\frac{2f}{r_{m}}KG\left(z,t\right)$$

Changeinglucosemasswithtime=movementalongSIduetoadvection−Absorptionofglucose

Initial conditions:

(2) $$G\left(z,0\right)=\{\begin{array}{c} G_{0}\;for\;l_{0};\;\\ 0\;otherwise;\; \end{array}$$

Boundary conditions:

(3) $${\frac{\partial G}{\partial z}|}_{z=0}={\frac{\partial G}{\partial z}|}_{z=L}=0$$

Here G(z,t) is the glucose concentration at time *t*, and distance along the intestine *z,* and $\bar{u}$, is the mean velocity along the length of the intestine. The last term is the absorption of glucose, where K, is the mass transfer coefficient, *2/r*_*m*_ is the ratio of surface area to volume for a cylinder and *f* represents the increase in absorptive surface area due to the folds of the intestinal wall. It is assumed that the volume input is a bolus and enters the small intestine at position *l*_*0*_ from the entrance, which is equal the radius of the bolus of entering liquid.

The overall mass transfer coefficient, *K,* will depend on the mass transfer within the lumen, through the epithelium layer and into the blood (Tharakan et al., 2010). As we are mainly interested in bioaccessibility we will simplify the phenomena and will ignore the effect of transport through the epithelium and blood assuming they are rapid and not rate limiting (Bastianelli et al., 1996, Stumpel et al., 2001, Wang et al., 2010). Therefore mass transfer coefficient, *K* is calculated from the relationship between Sherwood (Sh=Kd/D), Reynolds ($Re=\rho \bar{u}d/\mu$) and Schmidt (Sc=μ/ρD) numbers, where *d* is the mean intestinal diameter, *L* is the length of the intestine, *D* is the diffusivity, *ρ* is the density, and *μ* is the viscosity. The flow is in the laminar regime (for a water like solution, Re ∼ 3), and the following empirical relationship is used (Carbonell, 1975).

(4) $$Sh=1.62\;Re^{1/3}Sc^{1/3}{\left(\frac{d}{L}\right)}^{1/3}$$

Rearranging in terms of *K* gives:

(5) $$K=1.62{\left(\frac{\bar{u}D^{2}}{Ld}\right)}^{1/3}$$

The diffusivity is calculated from the Einstein-Stokes equation, which will depend on the viscosity of the system:

(6) $$D=\frac{K_{B}T}{6\pi \mu r_{0}}$$

Here *K*_*B*_ is the Boltzmann constant, *T* is the absolute temperature (310 K), and *r*_*0*_ is the radius of the diffusing molecule.

Therefore the only parameter that we can control and manipulate will be the viscosity of the food, and from this we can manipulate the mass transfer rate. The mass transfer rate will be inversely proportional to the viscosity to the power of 2/3 i.e.:

(7) $$K\propto \frac{1}{\mu^{2/3}}$$

Looking at the effect of protrusions on the surface of the intestine, it can be approximated that the villi increase the surface area by around 10 times relative to a cylinder and the microvilli by around 20 times (Ganong, 2005, Stoll et al., 2000). But only around 2% of the surface will be involved with the absorption of glucose, due to the fast speed of the absorption (Lentle and Janssen, 2011), giving an increased surface area of 4 times that of a cylinder. Including the effect of increased surface area from the presence of plicae circulares estimated at 3 times (Ganong, 2005, Stoll et al., 2000), this will give a value of *f* as 12. Values for parameters used in the models are shown in Table 2.

The results, typically of glucose absorbed can also be described in terms of calories where 1 g of glucose is 4 kcal.

The equation can also be made dimensionless, glucose was expressed as a fraction of the inlet concentration (*G’* = *G*/*G*_*0*_), time was divided by the residence time to give *τ* (=*t u/L*), and the distance along the intestine was divided by the length to give ξ (=*z/L*):

(8) $$\frac{\partial G'\left(\text{ξ},\tau \right)}{\partial \tau }=-\frac{\partial G^{'}\left(\text{ξ},\tau \right)}{\partial \text{ξ}}-\tau_{transfer}G'\left(\text{ξ},\tau \right)$$

Where,

(9) $$\tau_{transfer}=\frac{2fK}{r_{m}}\frac{L}{\bar{u}}$$

This yields the dimensionless number $\tau_{transfer}$ which is the characteristic time of mass transfer, i.e. the mass transfer rate (*2fK/r*_*m*_) multiplied by the mean residence time of passage through the small intestine (*L/*$\bar{u}$).

### 2.2 Model 2: stomach emptying and intestinal absorption of glucose

The gastric empting rate is thought to be the controlling mechanism in absorption of nutrients (Mourot et al., 1988, Hellstrom et al., 2006); for this reason a model was built to estimate the overall effect of the gastric emptying and mass transfer of glucose in the small intestine.

This model will treat the stomach as a reservoir for delivery of nutrients to the intestine only and will not consider its effect on the structure (chemical or physical) of the food. Gastric emptying is modelled as exponential decay, i.e. a liquid solution with no lag phase (Calbet and MacLean, 1997, Hellstrom et al., 2006), as this model shows a good approximation of the emptying for liquid only meals. The model for the intestine will be the same as for model 1 but with an input from the gastric emptying.

The glucose mass in the stomach was represented by G_S_:

(10) $$\frac{\partial G_{s}}{\partial t}=-\gamma G_{s}$$

(11) $${G_{s}|}_{t=0}=G_{s0}$$

The model for the small intestine will take the following form:

(12) $$\frac{\partial G\left(z,t\right)}{\partial t}=\{\begin{array}{c} \gamma G_{s}-\bar{u}\frac{\partial G\left(z,t\right)}{\partial z}-\frac{2f}{r_{m}}KG\left(z,t\right),\;\;if\;z=l_{0}\\ -\bar{u}\frac{\partial G\left(z,t\right)}{\partial z}-\frac{2f}{r_{m}}KG\left(z,t\right),\;\;\;\;otherwise \end{array}$$

(13) G(z,0)=0;

And the boundary conditions:

(14) $${\frac{\partial G}{\partial z}|}_{z=0}={\frac{\partial G}{\partial z}|}_{z=L}=0$$

Where G_S0_ is the initial input of glucose to the stomach (50 g) and, γ, is the decay constant, which can be expressed as the half-time of emptying, which is a common parameter used to describe the emptying of liquids from the stomach:

(15) $$t_{1/2}=\frac{\text{ln}\left(2\right)}{\gamma }$$

The model can also be made dimensionless in the same way as the advection-reaction equation to give:

(16) $$\frac{\partial G_{s}^{'}\left(\tau \right)}{\partial \tau }=-\tau_{empting}G_{s}^{'}\left(t\right)$$

Where,

(17) $$\tau_{emptying}=\gamma \frac{L}{\bar{u}}$$

Here $\tau_{emptying}$ is the characteristic time of gastric emptying and represents the rate of gastric emptying against the residence time in the small intestine. The half emptying times were varied between 10min and 3 h, which is within the range of typical values (seen in Table 1). The characteristic time of emptying was varied between 0.5 and 100, and the characteristic time of mass transfer was varied between 0.1 and 100, to see the effect on the fraction of glucose absorbed after the time is equivalent to the residence time.

### 2.3 Model 3: starch hydrolysis

In this work we will assume the starch remains intact until it reaches the small intestine, at which point hydrolysis of the starch, following Michaelis–Menten kinetics, will occur and a mass balance on starch and glucose in the small intestine has been carried out. In the small intestine model á-amylase will be in excess, and the ability to hydrolyse will be limited by the bioaccessibility of enzyme to starch, hence will be limited not by amount of enzyme but by the properties of the chyme (Ballance et al., 2013, Englyst and Englyst, 2005). The effect of salivary α-amylase is not included as the focus of the study is the hydrolysis in the intestine and hence the input is starch only into the stomach at t = 0. The model will therefore take a form similar to model 2, with an extra component of starch:

(18) $$\frac{\partial S_{s}}{\partial t}=-\gamma S_{s}$$

(19) $$\frac{\partial S\left(z,t\right)}{\partial t}=\{\begin{array}{c} \gamma S_{s}-\bar{u}\frac{\partial S\left(z,t\right)}{\partial z}-\frac{V_{max}S\left(z,t\right)}{K_{m}+S\left(z,t\right)},\;\;if\;z=l_{0}\\ -\bar{u}\frac{\partial S\left(z,t\right)}{\partial z}-\frac{V_{max}S\left(z,t\right)}{K_{m}+S\left(z,t\right)},\;\;\;\;otherwise \end{array}$$

Changeinstarchmasswithtime=movementalongSIduetoadvection–starchconvertedtoglucose

(20) $$\frac{\partial G\left(z,t\right)}{\partial t}=-\bar{u}\frac{\partial G\left(z,t\right)}{\partial z}+\frac{V_{max}S\left(z,t\right)}{K_{m}+S\left(z,t\right)}-\frac{2f}{r_{m}}KG\left(z,t\right)$$

Changeinglucosemasswithtime=movementalongSIduetoadvection+generationofglucose–absorbedglucose

Initial conditions and boundary conditions are the same as model 2, with input of starch.

These equations can be made dimensionless:

(21) $$\frac{\partial S^{'}\left(\text{ξ},\tau \right)}{\partial \tau }=-\frac{\partial S^{'}\left(\text{ξ},\tau \right)}{\partial \text{ξ}}-\frac{\tau_{R}S^{'}\left(\text{ξ},\tau \right)}{K_{mII}+S^{'}\left(\text{ξ},\tau \right)}$$

(22) $$\frac{\partial G^{'}\left(\text{ξ},\tau \right)}{\partial \tau }=-\frac{\partial G^{'}\left(\text{ξ},\tau \right)}{\partial \text{ξ}}+\frac{\tau_{R}S^{'}\left(\text{ξ},\tau \right)}{K_{mII}+S^{'}\left(\text{ξ},\tau \right)}-\tau_{transfer}G'\left(\text{ξ},\tau \right)$$

This yields two more dimensionless numbers as well as$\;\tau_{transfer}$:

(23) $$\tau_{R}=\frac{L}{\bar{u}}\frac{V_{max}}{Gs0}$$

(24) $$K_{mII}=\frac{K_{m}}{Gs0}$$

$\tau_{R}\;$ is the characteristic time of reaction, which is the residence time in the small intestine multiplied by the maximum reaction rate scaled with the initial input of starch.$\;K_{mII}$ is the Michaelis constant normalised with the initial input of starch.

The characteristic time of reaction will be varied from 1 to 25 as the characteristic emptying was varied between 0.5 and 100 and characteristic time of mass transfer was varied between 0.1 and 100 to see the effect on fractional absorption of glucose after the time is equal to the residence time.

### 2.4 Simulations

The equations for each model were simulated in gPROMS (v.3.7.1); the partial differentials were solved using backward finite difference method. All models were simulated over a period of 3 h, similarly to what is used for glycaemic index measurements (Brouns et al., 2005, Wolever et al., 1991). Graphs were produced using MATLAB (R2014a).

## 3 Results & discussion

### 3.1 Model 1

**Fig. 3:**
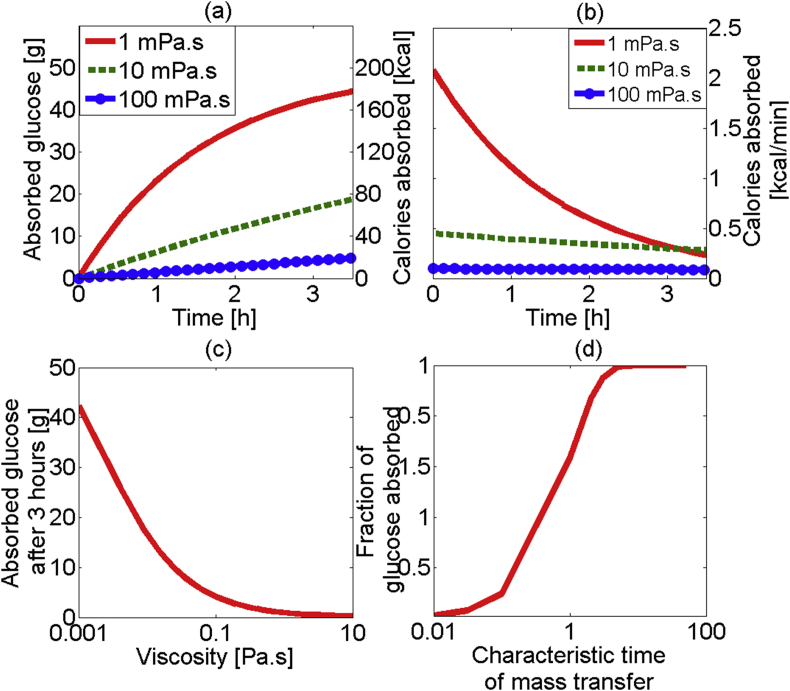
(a) the absorption curves for glucose solutions at different viscosities; (b) graph showing the total absorbed glucose after a 3 h period for solutions of different viscosities (log scale); (c) the fraction of glucose absorbed for the non-dimensionilised model against the characteristic mass transfer coefficient(log scale); (d) the rate at which calories are absorbed at different viscosities.

The first model investigated the mass transfer of glucose in the small intestine, from an initial input at t = 0 of 50 g of glucose solution at different viscosities (20 simulations for viscosities ranging 0.001 Pa s and 10 Pa s). The lower viscosity corresponds to viscosity of water, while the higher viscosity would be relevant to honey. Fig. 3(a) shows the amount of absorbed glucose against time. The initial rate of absorption decreases as the majority of glucose is absorbed, the effect being more pronounced at low viscosities. The results in terms of the rate of calories absorbed can be seen in Fig. 3(b). For low viscosities one can see an initial high rate of absorption as the luminal glucose is absorbed. From Fig. 3(a), it appears that by around 1 h, about half of the 50 g input of glucose has been absorbed and by around 3 h about 80% has been absorbed. This will result to a lower amount of glucose in the lumen, and a lower absorption gradient, which explains the decrease in the absorption rate for the low viscosity solution. When higher viscosity solutions were used the absorption rate stays almost constant over the 3 h simulated. This is due to the low mass transfer rate resulting in only a small percentage of the luminal glucose being absorbed. In Fig. 3(c) the effect of viscosity on absorbed glucose after 3 h is shown. Fig. 3(c) indicates that at low viscosities (1 mPa s) glucose is absorbed to a high extent (∼80% of input), and as the viscosity increases the amount of glucose absorbed decreases. For viscosity higher than 0.1 Pa s, the total absorbed glucose is less than 10% of the input and does not significantly reduce with viscosity. Fig. 3(d) is a dimensionless representation of Fig. 3(c), i.e. glucose absorbed versus viscosity/rate of mass transfer. The curve has a sigmoidal shape, showing a rapid increase between τ_transfer_ values of 0.1 and 3, corresponding to viscosity values of about 0.2 Pa s and 1 mPa s.

At low values of τ_transfer_, i.e., rate of absorption slow compared to the residence time, little absorption of glucose occurs; as τ_transfer_ increases an increase in absorbed glucose is observed. As the value of τ_transfer_ reaches 1, i.e., the rate of mass transfer is similar to the rate of advection along the length of the intestine, a plateau occurs with total absorption of the fed glucose.

These results indicate that the mass transfer coefficient within the lumen (determined by luminal viscosity) may have a large effect on the absorption of nutrients in the small intestine especially when mass transfer is significantly limiting the rate of absorption typically at viscosity values greater than 0.1 Pa s. Similar relationships have been seen *in vivo*, for example, Ellis et al. (1995) showed a non-linear relationship between zero shear viscosity of the chyme (measured in the jejunum) and absorption of nutrients in pigs, also indicating an inverse linear relationship between absorption over a 4 h period and concentration of guar gum in the meal (Ellis et al., 1995). Takahashi et al. (2009) showed how the disappearance of glucose in the small intestine of rats is inversely proportional to the viscosity (Takahashi et al., 2009), in this work 3 different viscosities were used and so there is not enough evidence to extrapolate these models. Fitting these results to a power–law curve the absorption was found inversely proportional to the viscosity to the power of around 0.45; this is lower than the relationship suggested in the present model (see equation (7)). This difference could be due to the added motility of a functioning gut, when digesting materials of high viscosity which is not included in this model. Due to secretions in the stomach and intestine the viscosity is unlikely to be constant with time, which is another limitation of the current model, but the trends here are consistent with those reported in the literature. Leclere et al. (1994) took a different view and speculated that the observed changes in blood glucose etc. from different viscosity meals are mainly due to the effect of the viscosity on stomach emptying rather than any mass transfer resistance within the small intestine (Leclere et al., 1994), which the current results show. Model 2 will be used to compare the emptying rate and mass transfer rate to test this hypothesis.

Overall in this work the parameters used were obtained from literature and the results were within the range of order of magnitude seen with *in vivo* data from literature see for example Dalla Man et al. (2006). Validation of similar *in silico* digestion models can be challenging as availability rather than postprandial glucose data would be required. As part of on going work we are aiming to make best use of existing *in vivo* data in the literature to validate our models. *In vitro* studies though demonstrate some agreement with the results presented here. Tharakan et al. (2010) showed a decrease in absorption with viscosity, pointing to an increase in diffusion resistance or decreased mixing efficacy as an explanation (Tharakan et al., 2010). A 50% decrease in absorption was seen when the guar gum was added at 0.5% compared to a starch mix with no guar gum. Gouseti et al. (2014) showed similar results for the absorption of glucose *in vitro* from model solutions for a range of food hydrocolloids. Others show similar trends (Sasaki and Kohyama, 2012, Singh et al., 2010, Slaughter et al., 2002), whilst speculating that the viscosity modifiers may have additional effects on the digestion process such as encapsulation of starch molecules, thus reducing bioaccessibility (Sasaki and Kohyama, 2012), or direct inhibition of digestive enzymes (seen with Guar Galactomannan) (Slaughter et al., 2002). The results here seem to agree with many of the experimental observations but the present model does not include any mixing effects, e.g., via segmentation or peristalsis. These are likely to increase the mass transfer rate and hence increase the absorption rate (Gouseti et al., 2014, Tharakan et al., 2010), and subsequently this could reduce the effect of viscosity upon the absorption rate. In future work the effect of mixing could be included in the mass transfer coefficient by investigating how segmentation/peristaltic mixing will affect the empirical relationship between Sherwood, Reynolds, and Schmidt numbers.

### 3.2 Model 2

**Fig. 4:**
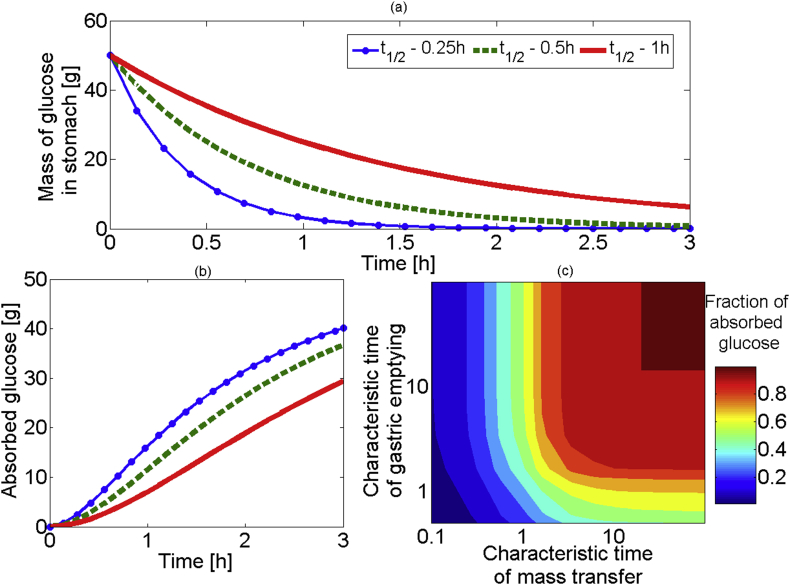
(a) mass of glucose in stomach over time with different half-time's of emptying and viscosity of 1 mPa s, (b) the absorbed glucose against time for 3 different gastric emptying half-time's, (c) contour plot of the characteristic mass transfer, against the characteristic emptying time on log–log scale, colour representing the fraction of glucose absorbed.

**Fig. 5:**
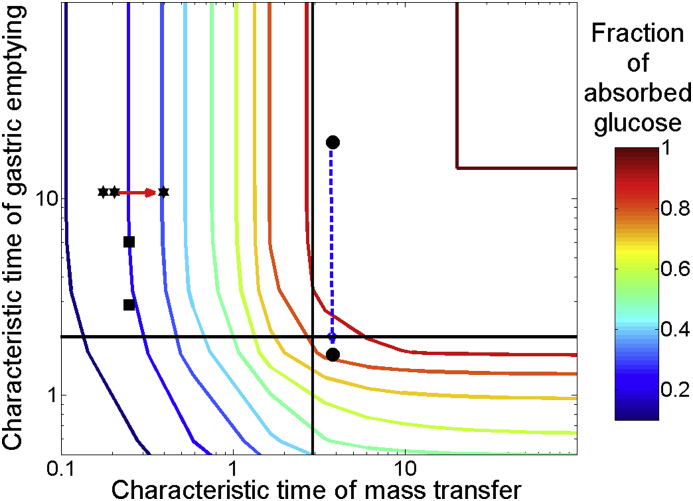
contour plot from Fig. 4(c) with plots from literature () (Marciani et al., 2000), (■) (Marciani et al., 2001), (●) from the model.

The second model expanded on model 1 by including the effect of gastric emptying, on glucose absorption. The input at t = 0 is into the stomach, and not into the intestine; the stomach then feeds the intestine. Fig. 4(a) shows the estimated emptying for glucose solutions for 3 different emptying half-times (15 min, 30 min and 1 h shown). Increasing the half-time results in a slower empting rate, as expected. Fig. 4(b) shows the associated glucose absorption in the small intestine. The total absorbed has a sigmoidal shape. For times smaller than 15mins the rate of absorption is low as expected from the small amount of glucose in the lumen (more than 50% glucose still been in the stomach). This is equivalent to an induction time. This is followed by an almost linear increase as more glucose enters the intestine and is available to be absorbed. The rate of absorption decreases after the majority of luminal glucose is absorbed. As the half-time of emptying increases, the induction time decreases and the rate of absorption decreases. Fig. 4(c) is a contour plot showing absorption of glucose versus emptying times (characteristic time of emptying) and viscosities (characteristic time of mass transfer). This plot can be separated into 4 regions: (1) the bottom right shows an area where the emptying rate is limiting, and greater characteristic time of emptying will result in greater absorption of glucose and vice versa; (2) the bottom left region shows an area where both emptying rate and mass transfer rate will be rate limiting; (3) the top left shows the area where mass transfer rate will be limiting only; and (4) top right area shows the area where near maximum absorption is reached (these regions are shown more clearly in Fig. 5).

The characteristic mass transfer time was varied from 0.1 to 100. The values of 0.1 and 3.4 corresponding to viscosities of 0.2, 10^−3^ Pa s, respectively; in this range of viscosities we would expect that mass transfer can be the rate limiting step. The higher values of characteristic mass transfer rates are in regime of effective and rapid mixing, i.e. one where mass transfer values are very large, e.g. K = 1 × 10^−6^ m/s The characteristic time of emptying was varied between 0.5 and 100; where the value of 3.2 and 100 resulted in half emptying time of 1 h and 2 min respectively. The lower value of 0.5 was included to investigate what happens for slow emptying and fast intestinal transit, corresponding to a 2 h emptying half-time and 1.5 h (Read et al., 1986) intestinal residence time.

In this work we will incorporate published research to understand the effect of gastric emptying (Brener et al., 1983, Calbet and MacLean, 1997, Marciani et al., 2000, Marciani et al., 2001, Shimoyama et al., 2007), using exponential decay to model the stomach emptying (Brener et al., 1983, Calbet and MacLean, 1997, Hellstrom et al., 2006). For reasons of simplicity the effect of secretions is not included in this model (Marciani et al., 2000, Marciani et al., 2001)

The two square data points (Marciani et al., 2001) represent low and high nutrient meals with similar viscosities. One would expect that they will have similar fractional absorption of glucose, even with a change in gastric emptying rate, as absorption is controlled from mass transfer. The majority of the data in Table 1 for liquid meals will appear into the upper left region of Fig. 5, indicating that the total absorption after around 3 h should be mass transfer limited.

The final set of points connected by a blue dotted arrow indicates how solutions, with similar initial viscosities (1 mPa s), would be affected by increasing the gastric emptying half-time from 10min to 2 h. In this case the system will see little effects in the total glucose absorption until it crosses the black horizontal line (around a half-time of 1 h). A further increase in the gastric emptying half-time will result in a shift to a gastric emptying limited region, which will cause a reduction in the fraction of glucose absorbed. Overall for relatively high viscosity food systems it appears that the fraction of glucose absorbed after 3 h is not controlled from gastric emptying rate, as with half-times of less than 90min (a characteristic time of emptying around 2), the system will be in the upper left region of Fig. 5, limited by the mass transfer coefficient.

### 3.3 Model 3

**Fig. 6:**
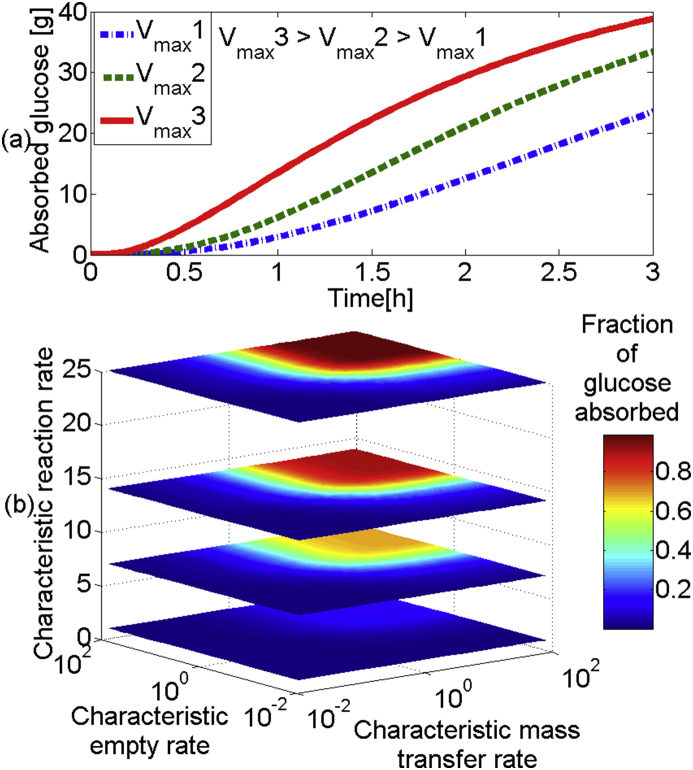
(a) absorption of glucose with time for systems with different starch hydrolysis rates (gastric emptying half-time 20min, viscosity = 1 mPa s, V_max_ = 4, 9 and 16 mmol/min), (b) contour plot showing the effect of gastric emptying rate, mass transfer rate and reaction rate for hydrolysis on absorption of glucose.

Model 3 incorporates the effect of starch hydrolysis to produce glucose on Model 2. In Fig. 6(a) absorbed glucose is plotted against time for different rates of hydrolysis, V_max_. One can see that the increasing V_max_ results in an increase in glucose absorption and decrease of lag phase (the initial slow absorption region). This is expected as the faster the starch is hydrolysed to glucose the faster glucose can be absorbed. However, increasing chyme viscosity will also affect the bioavailability of starch for reaction, or enzyme kinetics. In Fig. 6(b) contour plots of glucose absorbed for different characteristic reaction rates against characteristic emptying and mass transfer are shown.

The planes show similarities to Fig. 4(c), where the plot of characteristic emptying and transfer rate showed four regions. In Fig. 6(b), at low characteristic reaction rates there is little change in absorption as either emptying or mass transfer are changed, i.e., very little of the starch is hydrolysed to glucose, but increasing the reaction rate moves the system away from being reaction limited and the other parameters have a greater effect on glucose absorption, at around a characteristic reaction rate of 25, the starch is hydrolysed very quickly and behave similar to the Fig. 4(c), where the input is purely glucose. The two slices in the middle, V_max_ = 7.1 mmol/min (Satomura et al., 1984) and V_max_ = 14.1 mmol/min (Fonseca, 2011), show results for reaction kinetics taken from literature, and it can be seen that the reaction rate can be limiting if these rates are seen *in vivo*.

Each of these parameters is currently independent of the others, but in reality they are likely coupled. Changes in viscosity are likely to affect the emptying and mass transfer of nutrients as previously stated, as well as mass transfer of the enzymes. In addition, It is also important to consider other effects of food ingredients e.g., nutrient encapsulation by thickeners or direct enzyme inhibition by additives (Sasaki and Kohyama, 2012, Slaughter et al., 2002).

## 4 Conclusion

Mathematical models to describe *in vivo* digestion were developed and used to examine the relative effect of gastric emptying, mass transfer and reaction rate limitations in the small intestine. Within the assumptions of the models the results indicate that for gastric emptying half-times less than 1 h the viscosity/mass transfer rate is the limiting factor for the amount of glucose absorbed after 3 h. If the emptying half-time is greater than 1 h, both the gastric emptying and mass transfer rates can influence the absorption depending on the viscosity. If the mass transfer rate is faster than 1 × 10^−7^ m/s (i.e. luminal viscosity of 1 mPa s), the amount absorbed in 3 h is not limited by the mass transfer, and only by the gastric emptying rate. Starch hydrolysis reaction rates, when both the mass transfer and gastric emptying are fast and not limiting, can have a pronounced effect. The reaction kinetics for starch hydrolysis from literature showed around 25% difference in absorption when used in the model. Further development of the models is required to understand some of the controlling mechanisms as well as comparison with *in vivo* data to obtain confidence in the validity of the results.

## Figures and Tables

**Table 1:** Summary of gastric emptying data from literature, showing emptying rate for different liquid meals and the method of measurement, as well as comments to highlight the salient points of the studies.

Nutrient & thickener	Half-time [min]	Empty rate Kcal/min	Measurement method	Comments	Reference
500 mL 0.25 g/100 g LBG (μ_0_: 0.01 Pa s)	17 ± 6	–	Echo-planar magnetic resonance imaging	• No significant variation of emptying time with changes in viscosity • Large changes in viscosity occurred in the stomach, pointing to the importance of gastric secretions • Over 40min, the viscosity of the 0.01 Pa s solution was reduced to 0.005 Pa s and that of the 11 Pa s solution was reduced to 0.3 Pa s.	(Marciani et al., 2000)
500 mL 0.5g/100 g LBG (μ_0_: 0.1 Pa s)	18 ± 4	–
500 mL 1.0g/100 g LBG (μ_0_: 2 Pa s)	18 ± 7	–
500 mL 1.5g/100 g LBG (μ_0_: 11 Pa s)	19 ± 9	–
500 mL–64 kcal, LV	32 ± 7	1	Echo-planar magnetic resonance imaging	• Slowing of gastric emptying observed with addition of nutrient for both HV & LV • HV low calorie solution emptied slower than LV, the effect was diminished for high calorie solutions, but still significant. • Antral volumes were higher with HV meals compared to LV meals	(Marciani et al., 2001)
500 mL–64 kcal, HV	46 ± 9	0.7
500 mL–322.65 kcal, (63% lipid, 27% carbohydrate) LV	67 ± 9	2.4
500 mL–322.65 kcal, (63% lipid, 27% carbohydrate) HV	79 ± 6	2.0
600 mL–96 kcal Glucose LV	17 ± 1	2.8	Double sampling gastric aspiration technique	• Increase in emptying time (4–8 fold) with increased solution energy content (4 fold) • Show longer emptying for lower viscosity equicarbohydrate solutions, contrary to other authors.	(Vist and Maughan, 1995)
600 mL–96 kcal glucose HV	14 ± 1	3.4
600 mL–451 kcal glucose LV	130 ± 18	1.7
600 mL–451 kcal glucose HV	64 ± 8	3.5
600 mL- 60 kcal glucose solution	9.4 ± 1.2	3.2	Double sampling gastric aspiration technique	• Linear relationship between the caloric density and calorific empty rate was observed • Main factor in the emptying rate is the calorific density	(Calbet and MacLean, 1997)
600 mL- 132 kcal PPH	16.3 ± 5.4	4.05
600 mL–138 kcal WPH	17.2 ± 6.1	4.01
600 mL–396 kcal MP	26.4 ± 10	7.5
300 mL- 400 kcal glucose	107	1.9	Scintigraphy	• Solutions with high calories have longer emptying times • Solutions used varied in both volume and calorific content, hence making it difficult to identify the most important factor	(Phillips et al., 1991)
450 mL–200 kcal glucose	66	1.5
500 ml-500 kcal (mixed) LV	72.1 ± 19.5	3.5	Ultra-sonography	• with higher viscosity solutions having slightly longer emptying times • Results here show large variability (∼20–25%) • Calories are from mixed sources not just glucose	(Yu et al., 2014)
500 ml-500 kcal (mixed) HV	85.5 ± 16.5	2.9
400 ml- 400 kcal (mixed) LV	257.9 ± 31.8	0.8	^13^C breath sampling with continuous IR spectrometry	• Overall emptying faster for HV • Initial empty rate faster for LV • author linker this to inhibition due to nutrient sensing in the duodenum	(Shimoyama et al., 2007)
400 ml- 400 kcal (mixed) HV	195.1 ± 16.3	1.0
400 ml-Water	99.4 ± 2.8	–
240 kcal Solid/liquid meal	77 ± 6	1.56	Echo-planar magnetic resonance imaging	• Looked at effect of blended (soup) vs. Solid meal with water drink • Longer emptying for soup, linked by author to sieving mechanism whereby low nutrient liquid phase is able to empty separately from the high nutrient solid phase • The soup has homogenous nutrient composition and the emptying will stimulate the nutrient feedback mechanism, slowing the emptying rate.	(Marciani et al., 2012)
240 kcal Soup	92 ± 7	1.3

LBG – locust bean gum, PPH- Pea peptide hydrolysate solution, WPH- Whey peptide hydrolysate solution, MP- Milk protein solution, LV – low viscosity, HV – High viscosity, 1 g glucose = 4 kcal.

**Table 2:** Parameter values used in the model with references.

Parameter	Value	Reference
Surface area increase due to folds, villi & microvilli (*f*)	12	(Ganong, 2005, Stoll et al., 2000, Lentle and Janssen, 2011)
Mean velocity	1.7 × 10^−4^ m/s	(Stoll et al., 2000)
Length of small intestine	2.85m	(Stoll et al., 2000)
Radius of small intestine	1.8 cm	(Stoll et al., 2000)
Radius of glucose molecule (r_0_)	0.38 nm	(Schultz and Solomon, 1961)
Simulation time	10800 s	
Initial glucose/starch mass	50 g	
Viscosity	0.001–10 Pa s	
Emptying half time	2min – 2h	
V_max_	1-25 mM/min	(Fonseca, 2011, Satomura et al., 1984)
K_m_	9 mM	(Fonseca, 2011)

## Nomenclature

d: Diameter of intestine
D: Diffusivity
f: Increase in surface area due to intestinal wall protrusions
G: Glucose mass in intestine
G′: Dimensionless glucose mass in intestine
G_s_: Glucose mass in stomach
K: Mass transfer coefficient
K_B_: Boltzmann constant
K_m_: Michaelis constant
K_mII_: Normalised Michaelis constant ($K_{m}/Gs0$ )
L: Length of small intestine
R_0_: Radius of diffusing molecule
Re: Reynold number
r_m_: Mean intestinal radius
S: Starch mass in intestine
S′: Dimensionless starch mass in intestine
Sc: Schmidt number
Sh: Sherwood number
t: Time
t_1/2_: Gastric half emptying time
$\bar{u}$: Mean viscosity
V_max_: Maximum reaction rate
z: Distance along small intestine
γ: Gastric emptying decay constant
μ: Viscosity
ξ: Dimensionless distance along intestine
ρ: Density
τ: Dimensionless time
τ_emptying_: Characteristic emptying time ($\gamma L/\bar{u}$)
τ_R_: Characteristic time of reaction ($L/\bar{u}\;V_{max}/Gs0$)
τ_transfer_: Characteristic time of mass transfer ($2fK/r_{m}\;L/\bar{u}$)
